# A Few Days’ Obstetrical Experience

**Published:** 1879-02

**Authors:** R. W. Westmoreland

**Affiliations:** Washington, Arkansas


### A FEW DAYS' OBSTETRICAL EXPERIENCE.

By R. W. WESTMORELAND, M.D., Washington, Ark.

### QUININE AN ECBOLIC.

I have just been called in to attend two cases of miscarriage in which premature excitement was brought on by the administration of quinine, as the history of the two indicated to my mind.

Mrs. ——, æt 35, multipara, had been having chills for a day or two, when she took a "large dose of quinine" in the morning for the third chill. In about three or four hours she had a slight chill with consequent fever, and in a short time decided labor pains. These were only partially checked with opium and viburnum, and the following morning she gave birth to an eight months child.

But a day or two after the preceding, I was called hurriedly to see a lady who was threatened with miscarriage. She, too, had taken two or three "large doses of quinine" for the purpose of breaking the chills. The next day after taking the medicine labor pains commenced: for half a day and a night she was kept on opium and "black haw," with directions to let me know if they did not cease. I made known to the family that she would certainly miscarry if those remedies did not quiet the pains. The next morning they came, but being away from home that day the messenger did not find me; however, upon my next visit, she had so far recoverd as to lead me to feel no further uneasiness.

I believe it has been demonstrated by Hoffman, by experiment upon animals, that irritation of the hypogastric plexus produces motor activity of the circular fibres of the uterus. Upon this supposition the theory might reasonably be based that quinine, in addition to its general tonic action upon the spinal nervous centre, adds that property of being capable of so perturbing the normal equilibrium of involuntary functions as to bring about a deleterious termination.

HOURGLASS CONTRACTION.

Retained placenta I have always found to be not only troublesome, but critical, since the causes producing the difficulty are connected with a train of circumstances which presents a decided predisposition or actual existence of other and equally as grave complications. Whether the retention is occasioned by actual adhesions to the uterine walls, or from the utter inability of the uterus to throw off the mass (from an inherent or reflex incapacity), the general consequences amount to about the same, viz: free and often dangerous hemorrhage, inflammation and septicæmia from retained fragments of placenta, or blood-clots. Recurring attacks are often the sequel of these primary difficulties, until a predisposition to some puerperal exigency is almost erradically established. In the following case of hour-glass contraction, the history as obtained was in support of this view:

The lady had a miscarriage, and at her next pregnancy she underwent the trouble of removal of placenta. She described it as having to be "taken away by piecemeal." Two more miscarriages followed, with the placental difficulty in each, and then the fourth one, at which time I was called in. She had been having "chills and fever" for a day or two before, and when I saw her, was having labor-pains. I commenced giving her opium and virburnum until I got her quiet, when I left, with directions to keep the remedies up at regular intervals. Next morning the pains came on actively, and before I could get to her she gave birth to an eight-months fœtus. Living several miles off, and being very busy, I did not see her till three hours after the delivery. Up to that time she had had no after pain—no hemorrhage to amount to much—but complained of great prostration. Pulse was fast and feeble; pale, anxious countenance, and exhaustion much too excessive for only an hour's active labor. I administered brandy and ergot until I had given two good doses of each, when, no pain having made its appearance, after waiting a reasonable length of time, I made an effort to take the "after birth away" This I found impracticable from the fact that it was grasped firmly, about midway of its mass, by the band of uterine constriction. At first I was prone to believe that the os uteri had contracted from some local cause, (probably reflex sacral) but bi manual and rectal examination fortified me in the conclusion that I had to deal with the hour-glass trouble. Full doses of laudanum with whisky finally removed the spasm, and I took the placenta away with little trouble.

The uterus contracted down finely, and with the exception of being very weak, with slight fever, the patient did very well. Full doses of quinine were given every night for two or three days, with twenty-grain doses of bromide potash once each day for the same length of time, when the patient was pronounced well.

